# Editorial: At the top of the interneuronal pyramid—calretinin expressing cortical interneurons

**DOI:** 10.3389/fnana.2015.00108

**Published:** 2015-08-14

**Authors:** Filip Barinka, Zsófia Maglóczky, Nada Zecevic

**Affiliations:** ^1^Department of Neurology, University of RegensburgRegensburg, Germany; ^2^Institute of Experimental Medicine, Hungarian Academy of SciencesBudapest, Hungary; ^3^Department of Neuroscience, University of Connecticut Health CenterFarmington, CT, USA

**Keywords:** calretinin, interneurons, cortical development, epilepsy, primate cortex, corticogenesis, calcium-binding proteins

Intracellular protein calretinin (CR) acquired its name based on structural similarity to calbindin D28k and the site of first detection—retina (Rogers, 1987). Soon after this discovery, a basic description of the distribution of CR in rodent brain emerged from the work of several authors (reviewed by Baimbridge et al., 1992; Andressen et al., 1993). In the upcoming years, calcium-binding proteins (CaBP) calretinin, parvalbumin (PV), and calbindin (CB) could be shown to be expressed in largely non-overlapping interneuronal populations. The usefulness of utilizing PV, CB, and CR for categorization of interneuronal subpopulations was validated by gene cluster analysis (Toledo-Rodriguez et al., 2004).

While extensive literature on PV expressing (PV+) interneurons' physiological functions and changes in various pathological conditions have been published, we still have a limited view about the CR+ (and CB+) interneurons. This is quite disappointing, considering their increasing number in primates and various unique features they possess. In the Research Topic “At the top of the interneuronal pyramid—calretinin expressing cortical interneurons,” we intended to provide a summary of current knowledge on CR+ cortical neurons and especially to emphasize and discuss those “unique features” of CR+ neurons mentioned above.

In the first article of this compilation, the story begins with a mini-review on protein calretinin from Beat Schwaller (Schwaller, 2014). The Author not only offers a summary of what is known about the expression and physiological role of calretinin in cellular homeostasis, but, to mention one of many interesting aspects, the reader also learns why calretinin “may be a potential therapeutic target for treatment of Huntington disease” (see Dong et al., 2012 for original research report).

Further two original research papers focus on the development of CR+ neurons during the ontogenesis of human cerebral cortex. Miriam Gonzáles-Gómez and Gundela Meyer (González-Gómez and Meyer, 2014) put emphasis on the expression of CR in neuronal progenitors during the early period (between 6 and 14 gestational weeks, gw) of human corticogenesis. At the early embryonic time, the expression of CR is unrelated to the GABAergic neurons and could be seen also in glutamatergic neurons of the preplate and “pioneer” cortical plate. Complementary to this paper, Nevena V. Radonjić with colleagues (Radonjic et al., 2014) describes the pattern of expression of a transcription factor (Gsx2) and its two downstream effectors, (Ascl1 and Sp8) proposed to be involved in the CR cell lineage, in the human fetal cortex at later stadium of corticogenesis, at midgestation (20 gw). The authors reported the co-expression of these factors with CR+ cells that were dividing in the cortical ventricular and subventricular zones (VZ/SVZ). Their results support the hypothesis that the VZ/SVZ, besides the ganglionic eminences in subpallium, is an additional source of interneurons in the primate developing cortex. However, alternative hypothesis have been suggested (Hansen et al., 2013; Ma et al., 2013). In the next—4th article of our compilation, review paper from Ana Hladnik and colleagues (Hladnik et al., 2014), authors broadly discuss this controversial topic. They propose that contradictory results about the origin of cortical CR+ interneurons in primates might be partially explained by regional variations in the site of CR+ neurons origin. According to their hypothesis, the main source of CR+ interneurons for the frontal lobe might be the local pallial VZ/SVZ, while subpallial ganglionic eminences supply CR+ interneurons for posterior and lateral regions of primate cerebral cortex.

One fact, which indirectly supports the concept of an additional source of CR+ interneurons in primate cortex, is the significantly higher proportion of CR+ cells compared to non-primate mammals. Another two main classes of interneurons, those containing PV and CB (or somatostatin) do not show such a robust increase in proportion during evolution. In the 5th article of this Research Topic, mini review from Domagoj Džaja and colleagues (Džaja et al., 2014), authors address this topic and the significance of the 5-fold increase of CR+ neurons in the layers II/III of primate cortex in relation to rodents, for the function of neocortical neuronal circuits in primate brain. Importantly, they open and discuss the question of the role of those additional CR+ interneurons in formation of neuronal assemblies.

In the 6th article, Daniela Camillo with colleagues (Camillo et al., 2014) studies the visual response properties of neurons that express or have transiently expressed CR during their development in mouse primary visual cortex. Besides other interesting findings, their results again accentuate the (functionally not well understood) high extent of transient CR expression in pyramidal neuron precursors during corticogenesis.

Although the terms “interneurons” and “local circuits neurons” are often used synonymously, the CR+ interneurons were also shown to participate on long range cortical connectivity: by being targeted via connections from remote cortical areas (Gonchar and Burkhalter, 2003) as well as by sending long range cortico-cortical axons (Tomioka and Rockland, 2007). In the 7th article of our Research Topic, Rastislav Druga with colleagues (Druga et al., 2014) describes the distribution of CR+ neurons in the claustrum of the rat. Interestingly, a small portion of those neurons could be found to send dendrites in the neighboring telencephalic structures (striatum) or the insular cortex. This pattern of interconnectivity is consistent with the concept of claustrum as a global processing and synchrony detection system (Smythies et al., 2014), and implies that CR+ neurons could be directly involved in this function.

In the 8th article of our Research Topic, Bruno Cauli with colleagues (Cauli et al., 2014) provides a closing comprehensive summary of what is known about the anatomy and physiology of bipolar cortical CR+ interneurons. Besides the themes elaborated also in the individual articles of this compilation, additional exciting topics as for example the involvement of CR+ neurons in neurovascular coupling, are reviewed here. Summarizing the available evidence, authors state that probably the bipolar CR+ interneurons are amongst the most complex cells in the cerebral cortex because they seem to be involved in every major mechanism that supports successful neuronal computation. Hence, from this point of view, the heterogeneous population of CR expressing neurons really seems to stand “at the top of the interneuronal pyramid.”

And finally, in the last, 9th article of this research topic, a “window” to an important research field—function and malfunction of CR+ interneurons in various neuropsychiatric disorders—is being opened. In this review paper, Kinga Tóth and Zsófia Maglóczky (Tóth and Maglóczky, 2014) discuss the vulnerability of CR+ hippocampal interneurons to epilepsy. They summarize that the sensitivity of CR+ interneurons to epileptic activity plays an important role in the impairment of dendritic inhibition in epilepsy, with potential significant involvement in epileptogenesis. A different situation, with preservation of CR+ interneurons and loss of PV+ and/or CB+ interneurons could be found in other conditions as schizophrenia, bipolar disorder, and major depression (Beasley et al., 2002; Cotter et al., 2002; Sakai et al., 2008). It remains an exciting task for the future to establish a more detailed view of the role of CR+ interneurons in various pathological states.

All the aforementioned contributions are collected also in a single e-book. We hope this may be useful for researchers approaching this research field. More than anything else, we hope to inspire further research on this intriguing neuronal population and on its function and malfunction in neural circuits.

## Conflict of interest statement

The authors declare that the research was conducted in the absence of any commercial or financial relationships that could be construed as a potential conflict of interest.
